# The Contribution of Nano-Alumina to Ultra-High-Performance Cement-Based Systems

**DOI:** 10.3390/ma17164120

**Published:** 2024-08-20

**Authors:** Eirini-Chrysanthi Tsardaka, Evangelia Tsampali, Maria Stefanidou

**Affiliations:** Laboratory of Building Materials, School of Civil Engineering, Aristotle University of Thessaloniki, 54006 Thessaloniki, Greece; extsardaka@gmail.com (E.-C.T.); eva-tsa@hotmail.com (E.T.)

**Keywords:** cement, heat hydration, nano-silica, nano-alumina, nano-calcium oxide, fresh properties

## Abstract

In the last decades, nano-silica (NS), nano-alumina (NA), and nano-calcium oxide (NC) particles have been incorporated into cementitious materials, and it seems that each one of them contributes uniquely to the materials’ properties. This research explores the influence of each nanomaterial on the fresh properties of cement pastes and their compressive strength evolution over one year. Low proportions (1.5% by weight) of nanomaterials were added to cement pastes, and their fresh properties, such as heat of hydration and X-ray diffraction patterns in the first hours, were analyzed. The compressive strength and open porosity were also measured long-term. The acceleration of hydration heat in NA-cement pastes is linked to enhanced hydration product formation at early ages. Among the tested nanomaterials, NA increased compressive strength by 10% at later ages. Although the fresh properties of NC-cement pastes remained unaffected, their open porosity decreased by 54% at 28 days. In contrast, the increase in heat of hydration in NS-cement pastes did not result in significant strength improvement. Based on these findings, NA was selected for ultra-high-performance cement (UHPC)-based material use. Its incorporation not only preserved the ultra-high-performance (UHP) properties but also provided additional benefits such as an increase in compressive strength under a CO_2_ atmosphere. Through detailed analysis, this research establishes that nano-alumina incorporation optimizes the microstructural development and compressive strength of ultra-high-performance cement-based systems, presenting a novel advancement in enhancing the mechanical properties and durability of these materials under various environmental conditions.

## 1. Introduction

Ultra-high-performance concrete (UHPC) exhibits compressive strength superior to that of regular concrete. According to ASTM C1856, UHPC must achieve a compressive strength of at least 120 MPa [1]. UHPC is suitable for a wide range of applications where strength is critical, such as bridges, ultra-high-rise buildings, and areas prone to seismic events [2,3]. The exceptional properties of high-performance concrete (HPC) and UHPC systems are achieved through a combination of factors: reduction of water to binder (w/b) ratio [4], increasing mixing time [5], and using superplasticizer (SP) [6], all of which help to minimize capillaries and porosity. Additionally, fine aggregates [7] and silica fume [8,9] contribute significantly to the high performance of these concrete systems.

UHPC also demonstrates better ductility and elevated resistance to mechanical, thermal, and chemical stresses [10,11], compared to regular concrete. Due to its enhanced performance, using UHPC reduces the need for rehabilitation throughout the structure’s lifecycle. Its high strength-to-volume ratio enables the creation of thinner structures, which can be advantageous not only for rehabilitation but also for aesthetic purposes.

Recent advancements in UHPC development include the addition of additives to both enhance and maintain strength while reducing environmental impact and costs by decreasing the amount of cement needed [12]. Furthermore, research has focused on incorporating nanomaterials into the matrix to reduce microscopic pores and improve the density of UHPC [4,13].

Nanoparticles have contributed significantly to UHPC applications due to their filling effect and packing improvement [5,13]. Also, the manufacturing methods of nanomaterials contribute very little to carbon emissions, according to Liu et al. [14], so they are considered a sustainable option. After a comparison between carbon dioxide emissions cases, the authors concluded that the manufacturing of UHPC using nanomaterials instead of plain cement is of great significance to the reduction of the greenhouse effect contributing to durable and sustainable materials. 

Most literature on UHPC nanomodification focuses on the use of nano-silica (NS) and nano-calcium carbonate (NCaCO_3_) [15,16]. Studies have shown that only small amounts of nanoparticles are necessary to modify flowability effectively [4]. The incorporation of NS and NCaCO_3_ in UHPC has successfully achieved high compressive strength, with increases ranging from 3.0% to 15.7%, depending on the type and amount of nanoparticles used and the curing conditions [14]. In 2016, Shi et al. specified that NS increased compressive strength in the first 7 days, while NCaCO_3_ contributed to strength gains between 7 and 28 days [15].

Another approach with nano-alumina fibers has shown promising results in enhancing the mechanical performance of UHPC materials. In 2020, Sobolev et al. replaced silica fume with small amounts of nano-alumina fibers, suggesting it as a more economical alternative for UHPC-based materials [17]. In 2021, hydrophilic nano-alumina fibers were added to UHPC, leading to improved self-healing properties and mechanical performance [18]. The same year, Zhang et al. reviewed the effect of nanomaterials on the workability of HPC. Regarding nano-alumina powder, they noted that its high surface area increased the water-to-binder ratio (w/b), thereby reducing workability [3]. 

Several studies have also tried to analyze the effect of nano-alumina on ultra-high-performance cementitious materials. The study by Mohammad Reza Sharbaf et al. [19] concludes that incorporating 0.5% nano-alumina (NA) significantly enhances the compressive strength and reduces the permeability of high-performance concrete (HPC). Higher NA percentages, however, decrease compressive strength due to water absorption and agglomeration.

Farzadnia et al. [20] concluded that incorporating nano-alumina (NA) into high-strength mortars enhances mechanical properties and durability at elevated temperatures. XRD analysis showed a lower calcium hydroxide (CH) intensity with NA presence, indicating improved hydration. The optimal 1% NA increased compressive strength by up to 16% and enhanced residual strength by up to 800 °C. Higher percentages led to decreased strength due to agglomeration and water absorption issues.

The study by Hongyan Chu et al. [21] explored the effects of nano-alumina (NA) on ultra-high-performance concrete (UHPC). In the article, it is indicated that NA improves the microstructure by reducing the threshold pore diameter and porosity of UHPC. Compressive strength tests showed that NA enhances UHPC’s mechanical properties, with an optimal NA content of 1.0% yielding the highest improvements. Specifically, NA increased compressive strength by 4.08% to 20.58% and flexural strength by 7.38% to 16.87%. The study concludes that incorporating NA into UHPC significantly improves its mechanical properties and durability, making it a viable additive for advanced concrete applications.

The present research article evaluates the performance of cement pastes with cement I42.5N, along with low proportions of NS, NA, and NC, respectively, and incorporates the most suitable nanoparticles into UHPC. The study was conducted in two stages. During the first stage, cement pastes were produced adding different nanoparticles in 1.5% wt. During the fresh state of the pastes, the hydration heat was recorded while mineralogical analysis with XRD was also performed. In addition, the mechanical properties of the produced pastes were recorded at different ages. The results of the first state analysis were evaluated, and in the second state, NA was selected to continue the study (based on its ability to accelerate the hydration of cement and not interfere with the w/b ratio while contributing to the compressive strength development of the systems). Two different proportions of NA (1.5% wt and 3.0% wt) were added in the UHPC in order to examine the mechanical properties, flowability, and influence in the cement systems. 

## 2. Materials and Methods

### 2.1. Characterization of Raw Materials

Cement I42.5N was supplied from a local plant and the characterization is given in Table 1. The determination of metal oxides of cement was conducted with X-ray fluorescence, using the S8 Tiger instrument of Bruker (Karlsruhe, Germany). The particle size distribution analysis was performed using a Mastersizer 2000 instrument by Malvern Instruments. Ion Chromatography (IC) (Worcestershire, UK) was employed with a Dionex ICS-1100 instrument from Thermo Scientific Instruments (Waltham, MA, USA) to assess the water-soluble chloride and sulfate content. The eluent buffer consisted of 4.5 mM Na_2_CO_3_ and 1.4 mM NaHCO_3_, with a 1.2 mL/min flow rate and detection by suppressed conductivity. The mineralogical composition and the amorphous content were calculated with XRD instrumentation, specifically the D2 Phaser 2nd generation by Bruker Instruments (Billerica, MA, USA), with data analysis performed using EVA V5.0 software by Bruker. 

Nano-silica (7 nm), nano-alumina (<50 nm (TEM)), and nano-calcium oxide (<160 nm) were all 99.0% pure and were supplied in powder form by Sigma-Aldrich.

**Figure 1:**
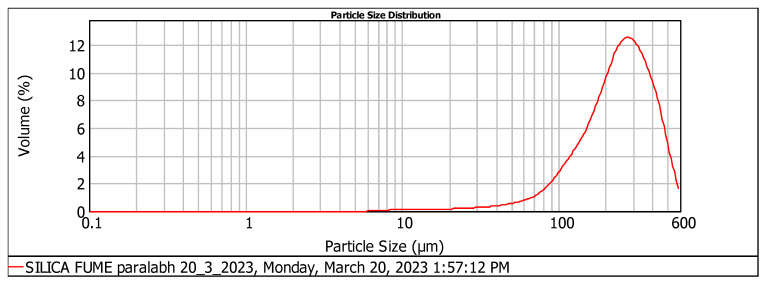
Particle size distribution analysis of silica fume.

**Figure 2:**
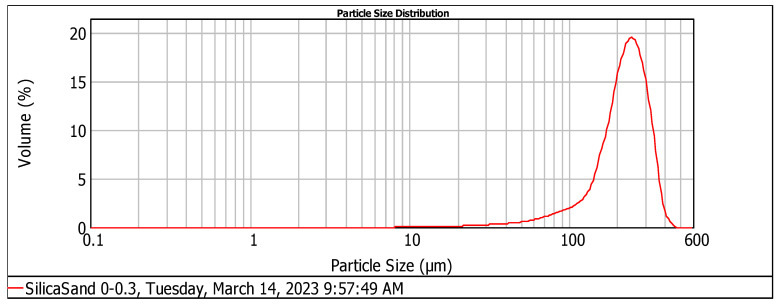
Particle size distribution analysis of silica sand.

The particle size of silica fume and silica sand was analyzed using the instrument Mastersizer 2000. According to Figure 1 and Figure 2 and Table 2, the silica sand had D (0.1), D (0.5), and D (0.9) values of 119.68 µm, 226.94 µm, and 320.67 µm, respectively. 

This fine silica sand helps to enhance the compressive properties of the material [22]. In contrast, the silica fume had D (0.1), D (0.5), and D (0.9) values of 107.96 µm, 249.90 µm, and 430.50 µm, respectively, indicating that the silica fume particles are coarser than those of the silica sand.

### 2.2. Preparation and Testing of Cement Paste Specimens

Cement powder was weighed and then mixed with the appropriate amount of water using a mechanical stirrer to achieve a regular consistency reference cement paste, following the guidelines of EN 196-3 [23]. The required amount of water was 310 g, which was also used for all the nano-modified systems. The nanoparticles (15 g) were added to the water before being added to the mixing bowl, based on previous experience [24], except for the NS particles, which were subjected to an ultrasonic bath. The pastes were cast into specimens with dimensions of 25 × 25 × 50 mm^3^. After one day, the specimens were de-molded and cured by water immersion until testing. The composition of the pastes is provided in Table 3. 

The measurement of hydration heat began immediately after determining the consistency of the fresh paste. The heat of hydration for the pastes was assessed using the TAM Air eight-channel isothermal calorimeter, TA Instruments in accordance with ASTM C1679 requirements. This device continuously monitors the heat evolution in pastes, with a tested sample volume of 5 mL. Data were recorded continuously for five days for each test, employing a data logger connected to a computer. A baseline was established within 12 h, and signal stability conditions were achieved using the linear least-squares procedure. The criteria established were an absolute value slope of the calorimetric signal below 3 µW/h and a standard deviation of less than 12 µW. 

The X-ray diffraction analysis was utilized to investigate hydration products with D2 Phaser 2nd generation Bruker Instruments. X-ray diffraction patterns were recorded at Cu Ka (30 kV and 10 mA, λ = 1.540 A) from 2° θ to 75° θ, with step 0.02° θ and time per step 0.4 s. EVA V5.0 software (Bruker) and COD (Crystallography Open Database) were used to identify XRD functions and diagrams. 

Compressive strength was measured according to EN 196-1 [25] using a computer-controlled WAW-300E Universal Testing Machine (China) at 7, 28, 90, 180, and 365 days. Open porosity was determined using the RILEM CPC 11.3 method in water under vacuum at the same testing ages. 

### 2.3. Preparation and Testing of High-Performance Concrete Specimens

Cement powder (I42.5N), silica fume, and silica sand were weighed and mixed with the appropriate amount of water using a mechanical stirrer for approximately 10 min. All the ultra-high-performance systems contained the same water-to-binder (w/b) ratio of 0.23 and the same amount of superplasticizer 3.0% (SP, polycarboxylic-based plasticizer). The composition of the systems is provided in Table 4. The concrete was cast into specimens with dimensions of 25 × 25 × 50 mm^3^. After demolding, the specimens were cured under water immersion until the day of testing. At the respective ages, the specimens were removed from the water and left to air dry for 6 h prior to compression testing and placed in an oven at 70 °C for 24 h before porosity determination. 

The compressive strength was measured according to EN 196-1 [25] with a computer-controlled WAW-300E Universal Testing Machine model at 7 and 28 days. Open porosity was determined according to the RILEM CPC 11.3 method in water under vacuum at the respective ages. 

The carbonation of UHPC specimens with and without nano-alumina was tested under two different curing regimes. After 28 days cured in water, the specimens (5 specimens of each synthesis) were placed in chambers. The conditions in the first chamber were; a temperature of 22 ± 2 °C and a humidity of 55%. The conditions in the second chamber were; a temperature of 24 ± 2 °C and a 3% CO_2_ atmosphere. The purpose of this testing was the verification of CO_2_ resistance and the evolution of compression in both curing regimes. The calcium carbonate (% wt) was quantified through Thermogravimetric Analysis measurements, using a NETZSCH STA 449 F5 Jupiter Simultaneous TG-DSC instrument, in an N_2_ atmosphere (50 mL per minute) from 50 °C to 1000 °C and 20 °C per minute step, at 7, 28, and 90 days. The quantified calcium hydroxide and carbonated species of the UHPC systems were made by utilizing the mass loss between 350 °C to 550 °C and 650–800 °C, respectively.

## 3. Results 

### 3.1. Heat of Hydration of Cement Pastes

**Figure 3:**
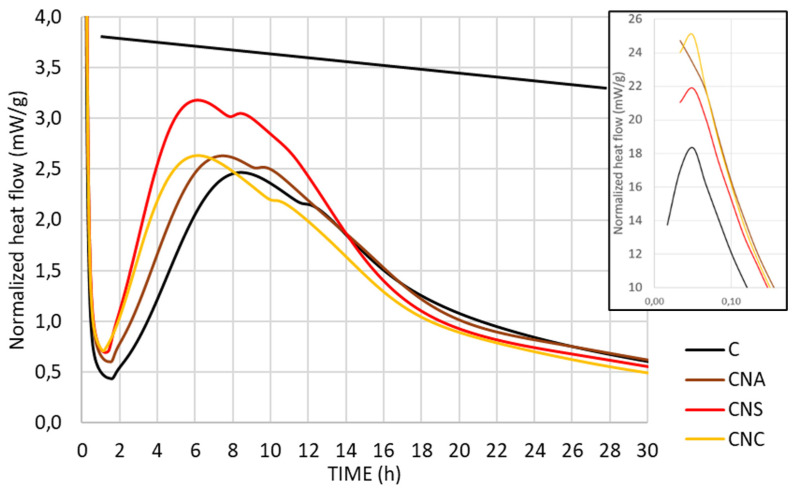
Isothermal calorimetric curves for the cementitious pastes and zoom in on the 1st hour of reaction on the top right.

**Figure 4:**
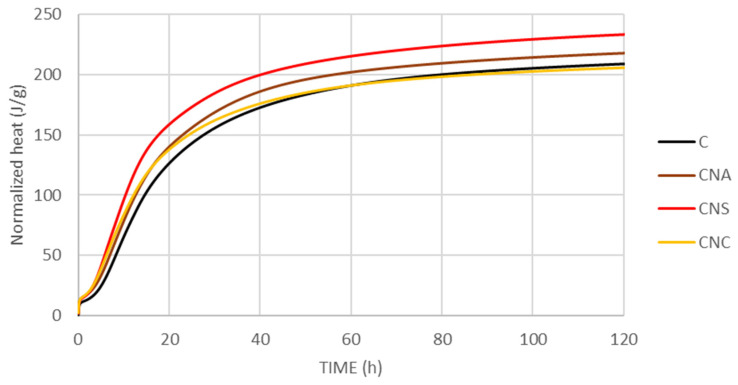
Normalized heat for the cementitious pastes.

The heat of hydration of all cement pastes was tested for 120 h. The results of the heat of hydration curves and the total heat obtained through isothermal calorimetric analysis are depicted in Figure 3 and Figure 4.

In the investigation of nano-alumina (NA) addition on Ordinary Portland Cement (OPC) hydration, normalized heat evolution curves revealed significant modifications in early-age kinetics. NA presence subtly enhanced the heat evolution rate during the induction period, indicating an early impact on hydration kinetics. More pronounced were the effects during the acceleration phase, where NA notably increased alite hydration rates, as evidenced by steeper heat evolution slopes and elevated peak rates around 7 h, compared to the reference that was at 8 h (Figure 3). This suggests an acceleration of strength development processes. Further, the addition of NA-amplified ettringite formation signals expedited the transition to the monosulfoaluminate (AFm) phase, indicating an influence on both silicate and aluminate hydration reactions. This has been observed in Figure 3, where a low, broad hump around 15 h in the heat flow curve of plain cement paste, as described, is associated with the transition from ettringite (AFt) to the monosulfoaluminate phase (AFm) [26,27]. The observed acceleration in alite and potentially aluminate reactions is attributed to the seeding effect of NA particles, providing more active nucleation sites for hydration products. Additionally, the high surface area of NA particles may increase sulfate ion absorption, decrease pore solution sulfate concentration, and accelerate monosulfate formation (Figure 3).

For CNS paste, isothermal calorimetric analysis revealed that nano-silica addition to cement paste enhances hydration heat and reaction rates. Notably, three distinct peaks characterize the hydration process, an initial peak within 0–1 h attributed to C_3_A hydration on cement particle surfaces, a second peak around 5–7 h resulting from C_3_S consumption and C-S-H and CH formation, and a third peak between 8–13 h associated with sulphate-type-AFm (monosulfate) formation, either from direct C_3_A hydration with solution sulfate or through secondary hydration involving ettringite (Figure 3) [28,29]. Increased nano-silica content correlates with much higher reaction rates, aligning with existing research. This acceleration is credited to the nucleation effect of nano-silica, acting as a fine filler to provide additional nucleation sites and potentially due to the shearing effect it introduces. Moreover, nano-silica reduces the induction period, with accelerated hydration observed at early stages compared to control samples, particularly as nano-silica content rises. 

The inclusion of NS not only increases the overall heat generated during hydration but also visibly accelerates the reaction rate (Figure 4). Additionally, it is noted that cement pastes with NS exhibit more heat release before 11 h and less after. The 11-h mark, referenced as the onset of the diffusion-controlled deceleration stage, marks a shift in hydration kinetics. These observations indicate that nano-silica, through its nucleation effect, acts as an effective filler material. This accelerates cement hydration by providing additional sites for nucleation, thereby facilitating the hydration of cement particles. Recent studies further suggest that this acceleration could also be due to a shearing effect introduced by the nano-silica particles, enhancing the mix’s reactivity [26].

The introduction of nano calcium oxide (NCaO) into the cement paste also accelerates the kinetics of early hydration, utilizing the high reactivity and the high specific surface area of NCaO. This acceleration is evidenced by increased early heat release rates observed in isothermal calorimetry studies, indicating a faster reaction rate and denser initial microstructure formation. Despite this rapid early activity, the overall degree of hydration remains unchanged (Figure 3), suggesting that while NCaO speeds up the initial stages of setting and strength development, it does not affect the longer-term hydration process (Figure 4).

However, the accelerated formation of a denser microstructure leads to an earlier shift to diffusion-controlled hydration, characterized by a slower rate of reaction. This suggests that the benefits of NCaO, such as improved early strength, come with a trade-off in the form of an earlier onset of slower hydration stages due to reduced permeability. Thus, while NCaO effectively enhances the early hydration kinetics, its impact moderates over time, illustrating the complex role of nano-additives in cement hydration processes (Figure 4).

### 3.2. Early Age Diffraction Patterns

**Figure 5:**
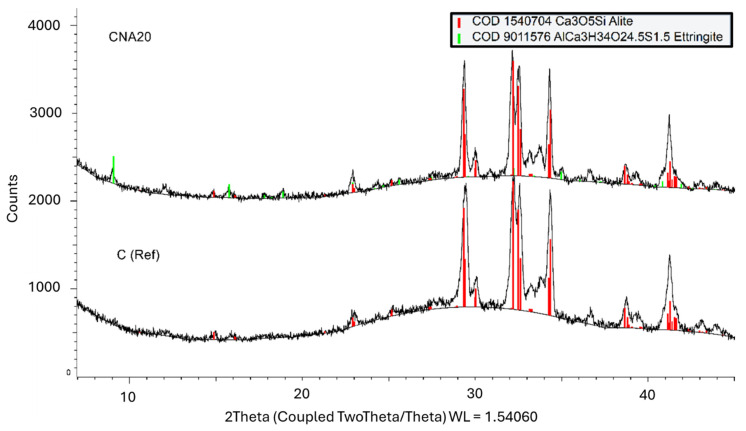
X-ray diffraction patterns of NA-modified cement paste with 20% wt NA, compared to plain cement paste, at 3 h.

**Figure 6:**
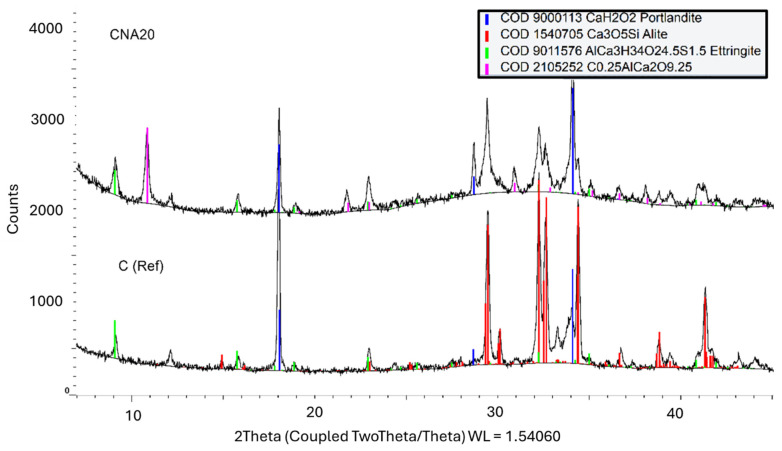
X-ray diffraction patterns of NA-modified cement paste with 20% wt NA, compared to plain cement paste, at 9 h.

The isothermal calorimetric curve of CNA demonstrates that NA contributed to the fastest hydration kinetics between the compared nanoparticles, from 0 to 0.10 h (Figure 3). Consequently, it is important to understand the early age formations and the additional compounds that are formed in the case of NA excess. The hydration of NA-modified paste was further investigated to verify the possibility of the enforcement of ettringite formation, as is mentioned in the literature, or new NA compound formation. A cement paste system was prepared with 20% wt NA content, only for analysis reasons to magnify the chemical reaction phenomena and not for mechanical properties investigation. This high NA content paste was compared with plain cement paste at the first hours of hydration. The comparison of X-ray diffraction patterns of these systems at 3 and 9 h is given in Figure 5 and Figure 6. According to the COD database, the identified mineralogical phases were alite, portlandite, ettringite, and AFm compounds. The alite phase is the main mineralogical phase of cement. In the first hours after mixing with water, alite peaks prevail in X-ray diffraction patterns of reference. More specifically, 3 h after mixing, reference systems contain only cement peaks, and CNA20 contains cement peaks (alite peaks) along with ettringite peaks, in agreement with the literature [27]. Nine hours after the hydration of cement, the hydration products were portlandite, ettringite, and AFM compounds, as depicted in the diagrams of Figure 6. In the presence of NA nanoparticles, after 9 h of hydration, an additional mineralogical phase was identified (COD 2105252). This compound is connected to calcium-aluminum carbonated compounds (Ca_2_Al(CO_3_)_0.25_O_9_) that NA is forming before leading to calcium carbonate, based on previous experience [28], or to ettringite and AFM phases enforcement. In this case, the proportion of NA is very high and unrealistic, but it demonstrates all the possibilities of NA action and was performed for research purposes. 

### 3.3. Compressive Strength and Open Porosity of Cement Pastes

**Figure 7:**
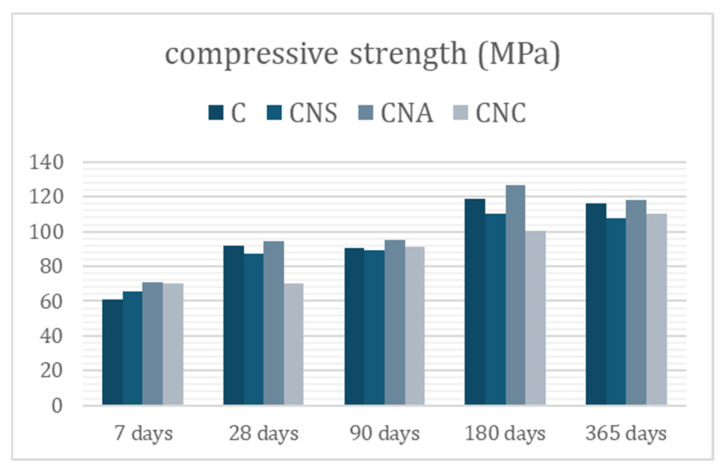
Compressive strength evolution of nano-modified cement pastes in time.

Compressive strength values (MPa) are given in Figure 7. The compressive strength of the reference system illustrates the behavior of plain cement. The incorporation of nano-silica (NS) nanoparticles did not significantly alter the compressive strength of the cement, with only a slight negative effect compared to the reference at all tested ages. Conversely, the addition of nano-alumina (NA) nanoparticles to the cement pastes primarily led to an increase in compressive strength. Notably, at the early age of 7 days, the NA-modified system showed a 15% increase in strength compared to the reference. At later ages, the improvement ranged from 1% to 6%. The incorporation of 1.5% by weight of nano-calcium (NC) in the cement paste resulted in a 14% increase in compressive strength at 7 days. However, at 180 and 365 days, the compressive strength decreased by 7% and 5%, respectively.

In agreement with the literature [29,30,31,32], the measured compressive strength values demonstrate that the incorporation of nanoparticles benefits cement-based systems at an early age, as evidenced by the 7-day strengths. The increase in early age strengths is likely related to the structure’s filling and the hydration acceleration when NS is added, as well as ettringite formation reactions when NA is added [24,33,34,35,36]. 

**Figure 8:**
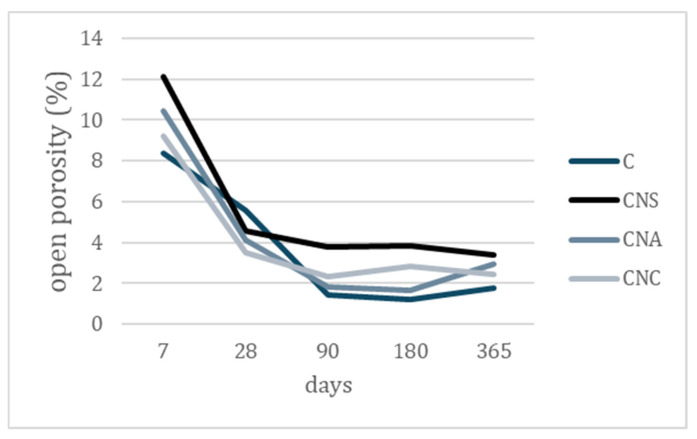
Open porosity evolution of nano-modified cement pastes in time.

The open porosity of cement pastes decreases over time, as shown in Figure 8, particularly at early ages. This decrease is due to the growth of calcium-silicate compounds, which reduces both the number and diameter of pores [37]. This trend is also observed in nano-modified systems. At 7 days, all pastes exhibit the highest values of open porosity, compared to the recordings of later ages. By 28 days, the porosity decreases, with nano-modified systems showing lower porosity than the reference. This result is consistent for all systems after 90 days, with small variations ranging from 1.8% to 4.0%. Among the nanoparticles, NS-modified pastes exhibit the highest porosity values, and reference systems along with NA systems exhibited the lowest values.

## 4. Discussion

Nano-particle additives such as nano-alumina (NA), nano-silica (NS), and nano-calcium oxide (NCaO) distinctly impact the heat of hydration in cement pastes, each through a different action path due to their intrinsic properties. NA accelerates alite hydration [17,26], leading to sharper early peaks in calorimetric analysis, primarily by providing nucleation sites. X-ray diffraction (XRD) investigations showed that the presence of NA could also trigger the formation of calcium-aluminum compounds, depending on the proportion of NA in the system. This not only accelerates hydration and enhances compressive strength but also contributes to the modification of porosity. NS significantly enhances early hydration rates through pozzolanic reactions with calcium hydroxide, forming additional calcium silicate hydrate and increasing paste density. This improves early strength but may slow down later hydration stages.

Conversely, NCaO markedly boosts early hydration kinetics without altering the cumulative heat released. This leads to a denser early matrix but potentially causes an earlier onset of slower, diffusion-controlled hydration phases. These nano-additives modify cement paste characteristics differently, highlighting their specific roles in optimizing cement hydration and microstructure development.

Nano-alumina (NA) was selected for incorporation into UHPC samples over nano-silica (NS) and nano-calcium oxide (NC) for two primary reasons. First, NA demonstrated the optimum compressive strength values over time (Figure 7), showing a positive influence on cement with small variations compared to NS. NA-modified systems consistently outperformed the reference, while NC-modified systems exhibited unstable behavior, often resulting in lower strength values than the reference. Second, the consistency of NA-modified pastes remained stable compared to the reference. NS-modified pastes showed a slight decrease in compressive strength and were the only ones to alter the paste consistency, although the values remained within regulatory limits (Vicat 6 ± 2 mm) [23]. 

**Figure 9:**
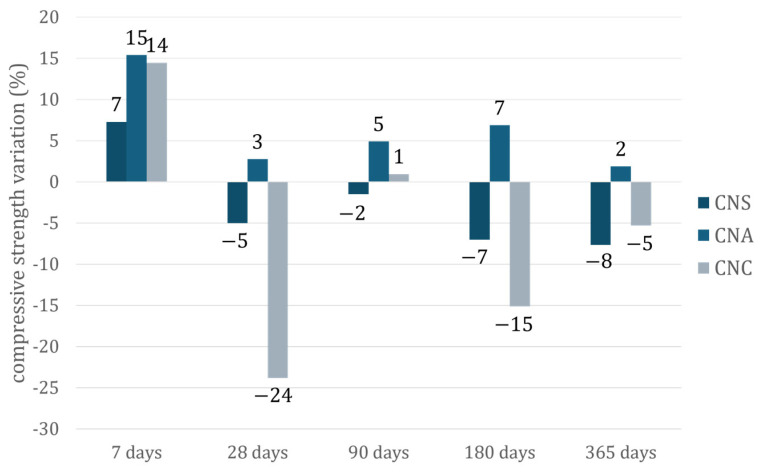
Variation of compressive strength (%) of nano-modified cement pastes compared to the reference cement paste, in time.

NS-pastes and NC-pastes were not chosen for UHPC applications in the present study due to two specific drawbacks. On the one hand, NS-pastes have a higher water demand than the other systems, which is discouraging for UHPC. Considering the significantly low w/b ratio that a UHPC system requires, NS addition would need further investigation. On the other hand, the addition of NC resulted in the lowest compressive strength trend between the pastes (Figure 9), although the decrease is below 25% in all tested ages. 

Consequently, in terms of the present study, NA seems a more suitable and efficient option, particularly in cases with low water-to-binder ratios, since NS tends to modify consistency and workability in cement-based systems (Table 5). Additionally, NA leads to favored interactions at an early age compared to NC addition. 

Furthermore, NS increased the early hydration rates through pozzolanic reactions [38,39,40], forming additional calcium silicate hydrate and enhancing paste density. This improved early strength, although later hydration stages showed slower progression. The inclusion of NS enhanced the heat of hydration but did not significantly alter compressive strength compared to the reference. NC significantly accelerated early hydration kinetics and initial microstructure formation, leading to improved early strength development. However, this rapid initial activity led to an earlier onset of slower diffusion-controlled hydration phases, limiting its long-term impact on compressive strength. NA demonstrated the most consistent improvement in mechanical properties over time, significantly increasing compressive strength by 15% at 7 days and 1–6% at later ages. It also reduced open porosity, resulting in denser and more durable cement pastes. An optimal NA content of 1.5% by weight was determined, providing the best balance of performance without adverse effects on workability or consistency.

**Table 5:** Why choose NA between the tested nanoparticles for UHPC applications?

NS	NA	NC
Reduces induction period and boosts early reaction rates	Accelerates early alite hydration and enhances sulfate ion absorption	Speeds up early hydration and microstructure formation
X	Early age ettringite support [27]	X
X	Early-age Ca-Al compounds support	X
Early-age C-S-H [41,42]	X	X
X	Carbonates [28]	Carbonates [43]
X	w/b	w/b
Increase compression only at an early age	Increase compression	Decrease compression

## 5. Ultra-High-Performance Concrete with NA

Among the tested nanoparticles, nano-alumina (NA) demonstrated a range of beneficial properties: stability in water consumption, consistent improvement in the mechanical properties of cement (I42.5N) over time, accelerated hydration, and enhanced hydration products (Table 5). 

The mixtures required more time than EN 196-3 suggested due to the low water-to-binder (w/b) ratio, with the total mixing time and manual handling extending to 10 min. The performance of NA was evaluated at 1.5% and 3.0% based on the weight of the cement. The addition of nanoparticles did not affect the mixing time, either positively or negatively. 

**Figure 10:**
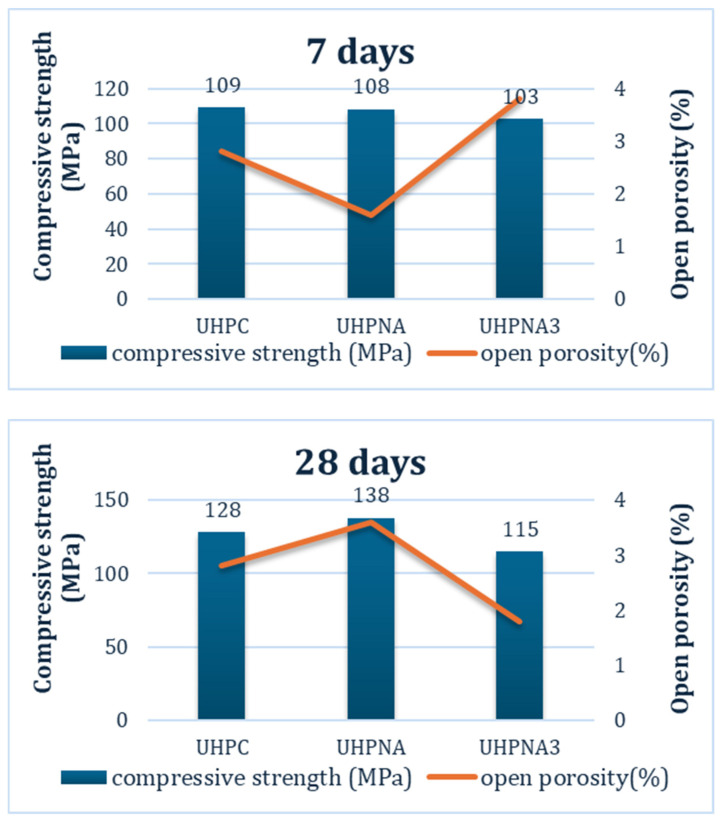
Compressive strength and open porosity of ultra-high-performance systems at 7 and 28 days.

The high compressive strength values are attributed to several factors: the ratio between the binder and aggregates (1:1), the type of aggregate (fine siliceous sand, 0–2 mm), the low water-to-binder ratio (w/b = 0.23), the high amount of superplasticizer (3.0% by mass of binder), the contribution of silica fume, and the compaction of the fresh material. The reference system (UHPC) achieved a compressive strength of 138 MPa at 28 days, surpassing the required value of 128 MPa for ultra-high-performance concretes (Figure 10).

Utilizing nano-alumina (NA) did not interfere with the water-to-binder (w/b) ratio, although it did modify the expansion of the nano-modified concretes. At a proportion of 1.5% by weight, the compressive strength (UHPNA) was maintained, while at 3.0% by weight, it fell below the threshold for ultra-high-performance concrete. Nonetheless, given that nanoparticles are recommended to be used in low quantities (below 3.0% by weight), ultra-high-performance concrete can benefit from the advantages of NA particles in powder form without compromising its mechanical properties. 

The ultra-high-performance (UHP) systems presented in this study, containing cement powder I42.5N, 15.0% by weight silica fume, fine silica sand aggregates, and 1.5% by weight nano-alumina (NA), complied with ASTM C1856. The proportion of NA did not significantly influence the compressive strength of UHP systems. However, the compressive strength values were around the regulation limit, with UHPNA achieving 138 MPa at 28 days and UHPNA3 reaching 115 MPa. An excess of nanoparticles typically reverses the benefits of compressive strength. In this case, 3.0% by weight NA is considered excessive, while 1.5% by weight NA is adequate. Additionally, using the proportion of 1.5% NA is more cost-effective compared to higher amounts of nanoparticles. This result indicates a catalytic role of NA in the systems [28], rather than contributing quantitatively. 

Many literature reports referred to workability decrease after NA incorporation in cement-based systems. Nazari et al. in 2010 [44] and Gowda et al. in 2017 [45] reported a reduction of workability by the increase in the percentage of NA. In 2020, Jiang et al. reported that NA addition reduced workability, and SP was used to maintain the w/b ratio and ensure that workability was uniform to all systems [46]. Sobolev et al. [17,47] and Muzenski et al. reported that surfactants might be absorbed and consumed to suspend the nanoparticles, so the workability in the presence of NA fibers in UHPC was reduced despite the addition of SP. In the mixtures of the present research, NA did not modify workability. The SP quantity used in UHPC, UHPNA, and UHPNA3 has contributed to the preservation of workability, regardless of the proportion of NA, 1.5% wt, and 3.0% wt, respectively. Considering that SP is necessary for the production of UHPC, to enable a low w/c ratio, NA could be used up to a proportion of 3.0% by mass of binder without significant workability affection. 

**Figure 11:**
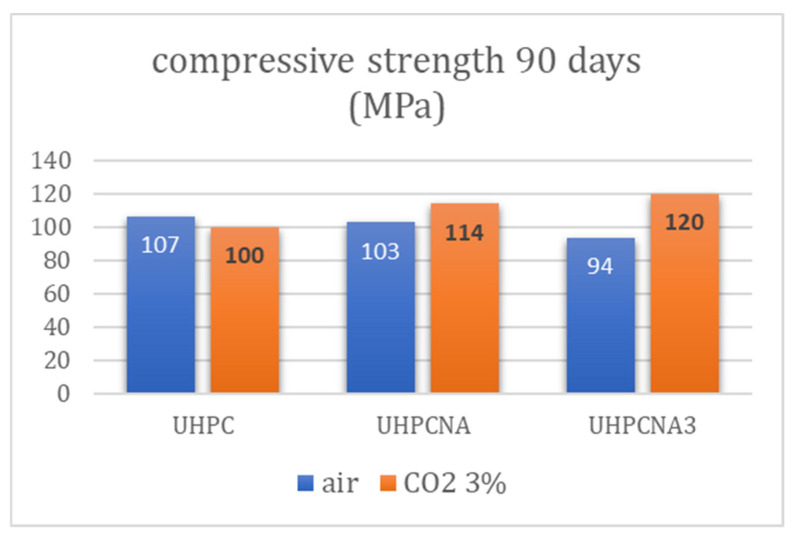
Compressive strength of ultra-high-performance systems after two different curing regimes at 90 days.

Although UHPC is not susceptible to carbonation [3] and long-term carbonation depth is not more than 2.0 mm in a 3% CO_2_ atmosphere [48], the specimens were tested in carbonation resistance. According to Figure 6, the NA additions could promote the carbonated species formation in cement-based materials, and also, according to our experience [28], these carbonated species could lead to calcium carbonate formation. It is also a fact that the curing influences the carbonation of the materials [49,50]. Figure 11 shows the evolution of compression of UHPC systems with the increasing NA proportion. The specimens cured on air presented a slightly decreasing compressive strength while the NA amount was increasing. In the case of UHPCNA, the decrease is less than 3.5%, but in the case of UHPCNA3, the decrease is 12.3%. On the contrary, the specimens cured in a 3% CO_2_ atmosphere presented enhanced compression by the increase of NA proportion. More specifically, UHPCNA and UHPCNA3 had greater compressive strength by 10.8% and 19.73% respectively, compared to the reference. As a result, it is demonstrated that in CO_2_-aggressive environments, NA additions in UHPC contribute positively to the mechanical performance of the systems. 

**Figure 12:**
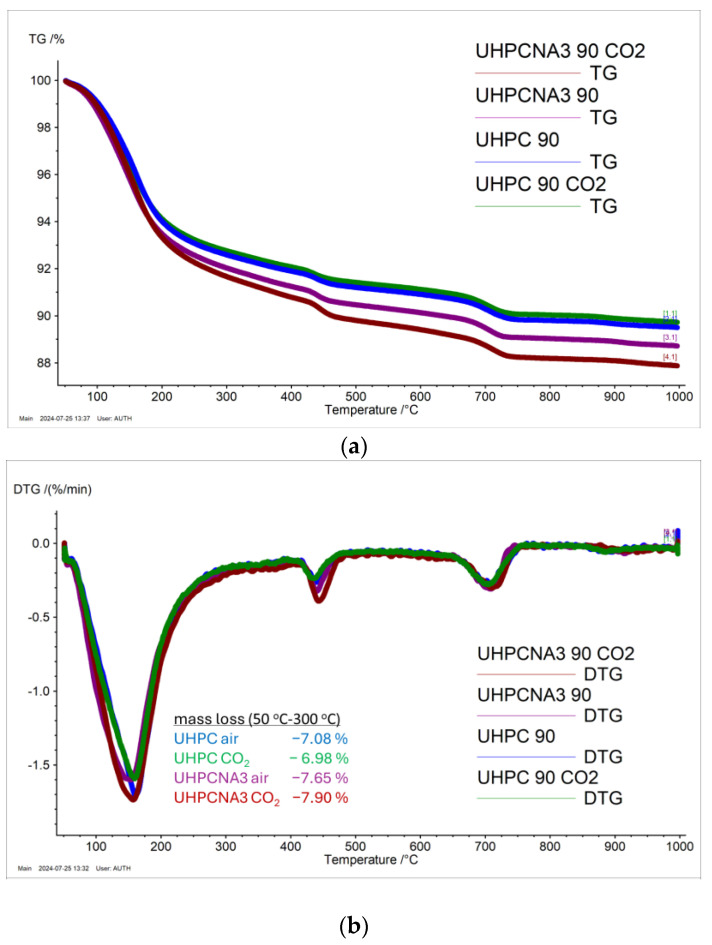
(**a**) TG thermograms and (**b**) DTG curves of ultra-high-performance systems after two different curing regimes at 90 days.

Natural carbonation of concrete takes a long time to exhibit [51] significant effects and it always happens slowly. Accordingly, the exposure of specimens to CO_2_ atmosphere could be considered to simulate aging. The NA addition did not influence the calcium carbonate formation in the CO_2_ atmosphere. As displayed in DTG curves (Figure 12), the decomposition of carbonates between 600 °C and 800 °C is similar for all the tested systems, regardless of the curing regime. The differences in mass loss between the systems are mainly between 50 °C and 300 °C, where AFM compounds and other aluminum-hydrated compounds lose their bonded water [52,53,54]. The mass loss increases with the addition of NA and the affection of carbon dioxide, but still, the modification is small. As a result, the addition of NA in UHPC systems is proven to resist carbonation and simultaneously present superior compression compared to the reference, under CO_2_ atmosphere. 

## 6. Conclusions 

The study evaluated the incorporation of nano-silica (NS), nano-alumina (NA), and nano-calcium oxide (NC) into cement pastes for comparison reasons to choose the optimum option for ultra-high-performance concrete (UHPC) applications. The study highlights the potential of nano-alumina to enhance both the performance and sustainability of ultra-high-performance concrete applications.

NS improved early age hydration and paste density; NC enhanced early strength and maintained consistency but had limited long-term effects; and NA offered the most comprehensive improvements in microstructural actions, mechanical properties, and durability prospects. The findings demonstrate distinct benefits of 1.5% wt nano-alumina emerging as particularly advantageous, mainly by combining superior mechanical performance, as well as micro-structure modifications that enforce the structure. The isothermal calorimetric curve of CNA demonstrates that NA contributed to the fastest hydration kinetics between the compared nanoparticles and also, diffraction patterns revealed additional calcium-aluminum compounds. Overall, NA-modified pastes maintained the w/b ratio and consistency of the pastes, as well as the workability measured in UHPC specimens, when at the same time exhibited the lowest porosity values and the highest compressive strength, achieving up to 138 MPa at the age of 28 days. 

It was verified that NA additions did not interfere with the carbonation resistance of UHPC systems, even under accelerated carbonation in the CO_2_ chamber. 

In humid conditions, NA 1.5% wt presented superior mechanical performance than NA 3.0%, demonstrating that NA possesses a catalytic role in the cement systems rather than contributing based on its quantity.

## Figures and Tables

**Table 1:** Characterization of cement I42.5N.

	Method	Cement Ι42.5 Ν
Density (g/cm^3^)	ASTM-C188-95	3.135
Cl^−^% wt	IC	<0.01
SO_4_^−2^% wt	IC	2.03
CaO%	XRF	61.7
MgO%	XRF	1.20
SO_3_%	XRF	3.34
Fe_2_O_3_%	XRF	3.07
Al_2_O_3_%	XRF	3.14
SiO_2_%	XRF	15.0
K_2_O%	XRF	1.10
Na_2_O%	XRF	-
TiO_2_%	XRF	0.02
Particle size	PSD	d (0.1): 2.438 μm d (0.5): 10.248 μm d (0.9): 28.661 μm

**Table 2:** Particle size in micrometers for silica sand and silica fume.

	D (0.1) (μm)	D (0.5) (μm)	D (0.9) (μm)
Silica sand	119.68	226.94	320.67
Silica fume	107.96	249.90	430.50

**Table 3:** Composition of reference and nano-modified cement pastes.

	Cement (g)	NS (% wt)	NA (% wt)	NC (% wt)	Water (g)	Consistency (mm) (6 ± 2 mm, EN 196-3)
C	1000	-	-	-	310	6.0
CNS	1000	1.5	-	-	310	8.0
CNA	1000	-	1.5	-	310	6.0
CNC	1000	-	-	1.5	310	6.0

**Table 4:** Composition of ultra-high-performance concrete specimens.

	Cement (g)	Silica Fume (g)	Silica Sand (0–3 mm) (g)	NA (% wt)	SP (% wt)	w/b	Workability (cm)
UHPC	850	150	1000	-	3.0	0.23	16.9
UHPNA	850	150	1000	1.5	3.0	0.23	16.3
UHPNA3	850	150	1000	3.0	3.0	0.23	16.5

## Data Availability

Data are contained within the article.
